# Prenatal X-ray exposure and childhood brain tumours: a population-based case–control study on tumour subtypes

**DOI:** 10.1038/sj.bjc.6604046

**Published:** 2007-10-30

**Authors:** K Stålberg, B Haglund, O Axelsson, S Cnattingius, S Pfeifer, H Kieler

**Affiliations:** 1 Department of Women's and Children's Health, Obstetrics and Gynecology, Uppsala University, Uppsala 75185, Sweden; 2 Centre for Epidemiology, National Board of Health and Welfare, Stockholm 10630, Sweden; 3 Department of Medical Epidemiology and Biostatistics, Karolinska Institutet, Box 281, Stockholm 17177, Sweden; 4 Rudbeck Laboratory, Department of Genetics and Pathology, Uppsala University, Uppsala 75185, Sweden; 5 Centre for Pharmacoepidemiology, Karolinska Institutet, Stockholm 17176, Sweden

**Keywords:** brain tumour, childhood, X-ray, epidemiology

## Abstract

We investigated childhood brain tumours by histological subtype in relation to prenatal X-ray among all children, less than 15 years of age, born in Sweden between 1975 and 1984. For each case, one control was randomly selected from the Medical Birth Register, and exposure data on prenatal X-ray were extracted blindly from antenatal medical records. Additional information on maternal reproductive history was obtained from the Medical Birth Register. We found no overall increased risk for childhood brain tumour after prenatal abdominal X-ray exposure (adjusted odds ratio (OR): 1.02, 95% confidence interval (CI): 0.64–1.62); primitive neuroectodermal tumours had the highest risk estimate (OR: 1.88, 95% CI: 0.92–3.83).

From 1975 to 2002, the incidence rate of childhood brain tumours (CBTs) in the United States increased from 2.3 to 3.5 cases per 100 000 children (NCI, 2005). A step increase was noted in 1984–1985, for which suggested reasons were better diagnostic procedures with MRI and changes in the histological classification (Smith *et al*, 1998). However, in a Swedish study between 1973 and 1992, the increase was around 2.6% annually, with no time clustering (Hjalmars *et al*, 1999). It is still unclear, therefore, whether the observed trend is a true increase or an effect of better diagnostic procedures.

Few risk factors are considered as established: rare genetic disorders and high-dose ionising radiation for cancer therapy and tinea capitis (Ron *et al*, 1988; Bunin, 2000; Baldwin and Preston-Martin, 2004). Radiological examinations during pregnancy have in some studies been associated with a slightly increased risk of CBT in offspring (Bithell and Stewart, 1975; Rodvall *et al*, 1990; Shu *et al*, 1994). However, meta-analyses have found no clear associations (Gurney *et al*., 1999; Bunin, 2000; Linet *et al*, 2003).

During recent years, more focus has been on risk factors for the subtypes of CBT, since their cellular origins as well as geographic distribution differ. The few previously existing studies on prenatal X-ray and subtypes of CBT are based on retrospectively collected information and exposure frequency was low (Bunin *et al*, 1994; Schuz *et al*, 2001).

The aim with this study was to assess any association between prenatal exposure to diagnostic X-ray and primary CBT in a population-based case–control study, including subanalyses according to histological subtype. To avoid selection and recall bias, we performed a nation-wide study, where information on X-ray had been prospectively recorded in antenatal records.

## MATERIALS AND METHODS

All children born between 1975 and 1984 in Sweden were eligible for this study. We used the nation-wide Swedish Cancer Register to identify children with brain tumours and for histological classification. The Cancer Register uses the 7th revision of the International Classification of Diseases (ICD-7) to facilitate comparison over time. Cases were children less than 15 years of age, diagnosed with brain tumour (ICD-7, code 193). The histological subtypes are defined through the three-digit pathohistological diagnosis code assigned by a pathologist. Controls were randomly selected children from the Medical Birth Register and frequency matched to cases by gender and year of birth. Controls had to be alive and residents in Sweden until the age of 15 years, which was ensured by using information from the Cause of Death Register and the Register of Population and Population Changes.

Medical information was abstracted from antenatal records, kept at local delivery archives and completed with data from the Medical Birth Register. Individual record linkage between registers and antenatal records was made possible by the Personal Identification Number (PIN), which is assigned to each Swedish resident and included in registers and medical records.

### Sources of data

The Medical Birth Register contains prospectively collected information on more than 99% of all births in Sweden since 1973 (Cnattingius *et al*, 1990). The information is provided through antenatal, obstetrical and neonatal records, which are filled in by midwives and physicians. The register includes data on maternal demographics, reproductive history and complications during pregnancy, delivery and the neonatal period. The register contains no data on X-ray examinations.

The Cancer Register was founded in 1958 and contains information about clinical and histological diagnosis and date and place of living at diagnosis. It is updated annually and has reliable information on more than 97% of all patients with cancer (Mattsson, 1977).

The Cause of Death Register contains data on dates and causes of death for Swedish citizens since 1961. The coverage is more than 99.5% and data are updated annually.

The antenatal records are kept in the hospital archives and include information on X-ray and ultrasound examinations and complement the Medical Birth Register in smoking habits, pregnancy symptoms and diagnoses.

### Study sample

A power calculation was performed in the planning phase of the study. By including 600 cases and an equal number of controls, and assuming a power of 80%, a two-sided 5% significance level and an abdominal X-ray exposure frequency of 10%, we should be able to detect an odds ratio (OR) of at least 1.7. In the Cancer Register, we identified 601 children born between 1975 and 1984 with a diagnosis of brain tumour before the age of 15 years. For 62 of the 601 cases, the PINs were incomplete or information on hospital of birth was missing, which made it impossible to identify their antenatal records. Of the remaining 539 cases, we were able to retrieve antenatal records for 512 (95%). For the 539 control subjects, we found 524 records (97%).

### Data collection and data management

Exposure data were extracted blindly with regard to case or control status from both antenatal records and registers. All data were assessed by one of the authors (KS) before transfer to the database. From the antenatal records, we included information on pregnancy complications and other current diseases and data on X-ray exposure. From the Medical Birth Register, we retrieved information on maternal age and reproductive history, gestational age at birth, birth weight and mode of delivery. Information concerning smoking habits was collected from the Medical Birth Register (data included from 1982) and completed by data from the antenatal records.

Radiological examinations were divided into abdominal and non-abdominal examinations. Abdominal examinations included pelvimetry, X-ray for foetal position and others. Risk for CBT was evaluated for all types of brain tumours combined and according to subtype. Each diagnosis was classified into one of the following subtypes: low-grade astrocytoma (astrocytoma grades I–II, benign opticusglioma), high-grade astrocytoma (astrocytoma grades III–IV), primitive neuroectodermal tumour (PNET) defined as category IC–D in the modified WHO classification by Rorke *et al* (1985), ependymoma (Rorke IA3, IA4), germ cell tumour (Rorke VI, VIId) or others (rare and not completely specified tumours).

We included information on numbers of X-ray examinations and the gestational week when they were performed. Gestational age was divided into trimesters: the first (gestational weeks 2–14), second (weeks 15–28), and third (weeks 29–45). Gestational age was calculated from data on last menstrual period, which was the standard procedure in Sweden at this time (Hogberg and Larsson, 1997). Birth weight and head circumference are presented as small, appropriate or large for gestational length, according to the gender-specific Swedish reference curves for birth weight and head circumference for gestational age (Niklasson *et al*, 1991).

### Statistical analyses

Logistic regression was performed to evaluate the association between prenatal exposure to diagnostic X-ray and incidence of CBT. Estimates of ORs and 95% confidence intervals (95% CIs) were calculated. In advance, we identified potential confounding factors that could interfere with both exposure and outcome, and for which it was possible to obtain information either from the registers or the antenatal records. The following confounders were included in the adjusted analyses: maternal age at birth, parity, multiple birth, mother's country of birth (Nordic country (Sweden, Norway, Denmark, Finland and Iceland) or non-Nordic country), hypertension during pregnancy, mode of delivery, breech position, gestational age at birth, birth weight, head circumference at birth and level of hospital (primary, secondary or tertiary level).

The statistical analyses were conducted with the SAS 9.1 software package.

The study was approved by the Ethics Committees at Karolinska Institutet and Uppsala University.

## RESULTS

In children with CBT, 50.4% were boys and 49.6% girls. As we matched for gender, the distribution between sexes among controls was similar. There were no differences in maternal smoking habits during pregnancy between cases and controls (36 *vs* 37% smokers, respectively). However, the information on smoking habits was missing for 36.5% of the sample. Further maternal and foetal characteristics are presented in Table 1. Among children with CBT, it was more common to be the first-born child (*P*=0.01) and to be born at a primary or secondary level hospital (*P*=0.04) than for controls. There were no other significant differences in maternal and neonatal characteristics between cases and controls (Table 1).

Overall, 21.1% (*n*=108) of the mothers to children diagnosed with CBT were exposed to X-ray during pregnancy compared to 21.2% (*n*=111) of mothers to the children randomly selected as controls. For abdominal X-ray, the exposure frequency was 10.7% (*n*=55) for cases and 9.4% (*n*=48) for controls. Beside pelvimetry and X-ray for foetal position, abdominal X-ray included three scintigraphy of placenta, four scintigraphy of kidney, four plain abdominal X-rays and one colon examination. For both cases and controls, more than 96% of the abdominal examinations were performed in the last trimester. Non-abdominal examinations included 107 pulmonary and one each of skeletal, sinus and dental X-ray. Only three individuals in each group were exposed to X-ray more than once during the pregnancy.

Table 2 presents the distribution of CBT subtypes and age at diagnosis. Median age for diagnosis for all CBTs was 8 years. For subtypes, ependymomas had the lowest median age at diagnosis (4 years) and high-grade astrocytomas had the highest (9 years).

In Table 3, the ORs for prenatal X-ray are presented by tumour subtype. Since there were no differences between cases and controls in frequency or timing of exposure, analyses are restricted to children to mothers exposed to prenatal X-ray, regardless of numbers of examinations or time of exposure. Being prenatally exposed to abdominal X-ray was not associated with an increased overall risk of brain tumours compared with being unexposed (adjusted OR: 1.02, 95% CI: 0.64–1.62). When stratifying according to histological subgroups, we found that PNET had the highest risk estimates (adjusted OR: 1.88, CI: 0.92–3.83). Astrocytoma low and high grade had no increased ORs (adjusted OR: 0.72, CI: 0.36–1.42 and adjusted OR: 1.06, CI: 0.39–2.86, respectively). As there were only 44 cases with ependymoma, it was not possible to perform multivariate analyses to adjust for possible confounders, and only crude risk estimates are presented. For similar reasons, germ cell tumours (*n*=17) were included in all cancers and not analysed separately.

## DISCUSSION

Prenatal X-ray exposure does not seem to increase the overall risk of CBT. However, for the subgroup of PNET, we found an increased OR of 1.88 (95% CI: 0.92–3.83). PNETs are found among the youngest children and is classified as an embryonic tumour. Medulloblastoma is the most common tumour in the PNET group, has a peak age of occurrence of 7 years of age and is located in the cerebellum. The other, and more rare tumours in the PNET group, may occur in the neonatal period (in rare cases congenitally), have a peak age at diagnosis of about 5 years of age and are mainly supratentorial (Sarkar *et al*, 2005).

It has been suggested that each cell type has a ‘window of vulnerability’ during which neoplastic transformation may occur (VandenBerg, 2001). Consequently, gestational age at exposure to X-rays might be of importance when evaluating risks for CBT. Since 96% of the abdominal X-ray examinations in this study were performed in the third trimester, it was not possible to evaluate the impact of time of exposure. However, the human brain grows and develops during the whole foetal period and there is currently no evidence that foetuses should be more sensitive to carcinogenic factors in the early gestational period.

In the present study, mean foetal absorbed radiation dose was hard to estimate since the X-ray examinations were not standardised and performed at several hospitals over a 10-year period. Foetal absorbed dose for an abdominal X-ray examination has been reported to vary between 0.1 mGy for a pelvimetry with one anterior–posterior and one lateral film (Axelsson and Ohlsen, 1979) to more than 4 mGy (Osei and Faulkner, 1999; ICRP, 2000). In dosimeter studies, radiation doses have been found to vary by a factor of 30 or more for the same type of examination depending on variations in radiological techniques, choice of film, etc, (Badr *et al*, 1997; ICRP, 2000). It is reported that prenatal exposure to a radiation dose of over 10 mGy may increase the risk of childhood cancer (Doll and Wakeford, 1997). Furthermore, it is suggested that there is no ‘safe’ threshold limit but instead a linear relationship between exposed dose and risk increase (Doll and Wakeford, 1997; National Research Council, 2006). Risk increases can be expressed as excess relative risk (ERR), and from previous studies of prenatal X-ray exposure, it is estimated that the ERR for childhood cancer for an absorbed dosage of 10 mGy is approximately 0.5 (Wakeford and Little, 2003). Although there are uncertainties concerning such risk estimates, the low radiation doses in the present study suggest that an ERR of more than 0.5 would not be expected.

The study had sufficient power to detect an OR of at least 1.7 for the overall risk of CBT. However, for the analyses stratified by histological subtypes, a larger population would probably have been needed to find statistically significant increased risks. For the subgroup PNET, which included 105 cases, we had a power of 52% to detect an OR of 1.9 at the 5% significance level. Since there are approximately 10–15 new cases of PNET among Swedish children annually, several more years had to be included to reach sufficient power. As the Medical Birth Register was founded in 1973, 2 years before the start of this study, this register could not have been used to reach the warranted number of cases with PNET, which would be needed to increase power. A study large enough to have sufficient statistical power for the subanalyses might be performed by pooling data from the Nordic countries all of which have population-based national registries and similar health-care systems.

The strengths of this study include the population-based design, the large number of cases of CBTs, the prospective and blinded data collection and the small number of missed cases. We were forced to exclude 5% of the cases and 3% of the controls, as we could not trace their antenatal records. Since the antenatal records are filled in and filled before any of the included cases were diagnosed with CBT, the exclusions should not have affected the risk estimates.

In two previous case–control studies on CBT subtypes, no increased risk after prenatal X-ray could be detected, neither for all CBTs nor for PNET specifically (Bunin *et al*, 1994; Schuz *et al*, 2001). In these studies, retrospective interviews on exposure data were used and the risk of recall bias could not be excluded. In both studies, less than 5% of the study population were exposed to prenatal X-ray, whereas we had an exposure rate of 21%. The present study is, to our knowledge, the first study on X-ray and CBT subtypes with prospectively collected information from antenatal records. The antenatal records in Sweden are standardised and are considered of high quality (Cnattingius *et al*, 1990).

Potential weaknesses are that some X-ray examinations may not have been noted in the records. However, we assume that such negligence was rare and we do not suspect that any possible misclassification of exposure would be different between cases and controls. Although we have performed multivariate analyses and adjusted for potential confounding factors, some other conditions that vary with both exposure and outcome could possibly have affected the results. Maternal smoking has, in some studies, been associated with a small increase in risk of CBT for the child (Brooks *et al*, 2004). However, as smoking was not included in the Medical Birth Register until 1982 and earlier data from the antenatal records were incomplete, information on smoking was missing for 37% of the study population. Therefore, it was not possible to include smoking in the adjusted analyses. On the other hand, for the 63% for whom we had information on maternal smoking; the distribution between smokers and non-smokers was similar between cases and controls. Furthermore, low birth weight is closely correlated to maternal smoking and adjustments for birth weight had only minor effects on the results.

Ultrasound has, during the last two decades, become the examination of choice for determining foetal status (position, multiple births, etc), and antenatal pelvimetry is rarely indicated. Nevertheless, abdominal radiation still occurs, for instance in CT scan on maternal indications. Risks with foetal diagnostic radiation could probably be extrapolated to the early childhood period, since the brain still develops and grows rapidly during the first years of life.

Our most important finding might not be the clinical implication, but in relation to the theory that prenatal or neonatal radiation might affect the various neural cell types differently. PNET are presumed to arise from undifferentiated neural stem cells and little is known regarding the molecular genetic events initiating carcinogenesis (Biegel, 1997). An interesting hypothesis would be that neural stem cells could be extra sensitive during their differentiation and that DNA damage caused by radiation could initiate malignant transformation, leading to the development of embryonic CNS tumours such as PNET. If an increased risk of PNET after radiation exposure is confirmed in future studies, this hypothesis could be tested further in laboratory or animal studies, as part of the complex work of mapping the pathogenesis of childhood brain cancer.

In conclusion, we found no increased risk for CBT after prenatal X-ray exposure when analysed as one group. When analysing according to subgroups, the embryonic tumours defined as PNET showed the highest risk estimates.

## Figures and Tables

**Table 1:** Maternal and infant characteristics for 512 children diagnosed with brain tumour and 542 children serving as controls

	**Childhood brain tumour**		**Controls**	
	***N***	**%**	***N***	**%**
*Maternal age* (*years*)				
15–24	178	34.8	167	31.9
25–34	305	59.6	320	61.1
35–44	29	5.7	37	7.1
				
*Birth order*				
1	245	47.9	210	40.1
>2	267	52.1	314	59.9
				
*Multiple birth*				
No	502	98.0	516	98.5
Yes	10	2.0	8	1.5
				
*Mother born in a Nordic country*				
Yes	491	95.9	496	94.7
No	21	4.1	28	5.3
				
*Gestational age at birth*^a^ (*weeks*)				
22–32	5	1.0	7	1.3
33–36	24	4.7	23	4.4
37–41	399	77.9	412	78.6
42–45	81	15.8	78	14.9
Missing	3	0.6	4	0.8
				
*Mode of delivery*				
Vaginal	425	83.0	422	80.5
Vacuum extraction	28	5.5	31	5.9
Caesarean section	59	11.5	71	13.5
				
*Breech position*				
No	503	98.2	513	97.9
Yes	9	1.8	11	2.1
				
*Birth weight, SDS* b				
SGA	9	1.8	12	2.3
AGA	477	93.2	493	94.1
LGA	19	3.7	14	2.7
Missing	7	1.4	5	1.0
				
*Head circumference, SDS* b				
SGA	15	2.9	13	2.5
AGA	473	92.4	487	92.9
LGA	19	3.7	13	2.5
Missing	5	1.0	11	2.1
				
*Level of hospital*				
Primary level hospital	161	31.4	150	28.6
Secondary level hospital	248	48.4	234	44.7
Tertiary level hospital	103	20.1	140	26.7
Total	512	100.0	524	100.0

AGA=appropriate for gestational age; LGA=large for gestational age; SDS=standard deviation score; SGA=small for gestational age.

a Calculated by last menstrual period.

b Standard deviation score, according to Niklasson *et al* (1991).

**Table 2:** Distribution of histological subtypes of childhood brain tumour and age at time for diagnosis

			**Age (years)**		
	***N***	**%**	**25th percentile**	**Median**	**75th percentile**
Astrocytoma low grade	191	37.3	4	8	12
Astrocytoma high grade	61	11.9	6	9	11
PNET	105	20.5	3	6	10
Ependymoma	44	8.6	1	4	9.5
Germ cell tumour	17	3.3	8	9	12
Others^a^	94	18.4	4	7	11
Total	512	100.0	4	8	11

PNET=primitive neuroectodermal tumour.

Age is presented as median age together with 25th and 75th percentiles.

a Two neurofibromas/hamartomas, seven meningiomas, one malignant meningioma, eight haemangioma, two hemangioblastoma, one lipoma, 23 craniopharyngioma, 32 tumours not specified as malignant or benign, one suspected malignant tumour, 17 tumours without pathohistological diagnosis.

**Table 3:** OR and 95% CIs for all CBTs combined and by brain tumour subtype in relation to type of prenatal X-ray exposure

			**Crude**		**Adjusted^a^**	
	**Type of X-ray**	**Exposed (*N*)**	**OR**	**95% CI**	**OR**	**95% CI**
All brain tumours^b^ (*n*=503)	Abdominal	51	1.07	0.70–1.62	1.02	0.64–1.62
	Non-abdominal	53	0.85	0.57–1.25	0.78	0.52–1.17
Astrocytoma low grade	Abdominal	15	0.83	0.45–1.53	0.72	0.36–1.42
	Non-abdominal	24	1.01	0.61–1.68	0.96	0.57–1.62
Astrocytoma high grade	Abdominal	6	1.00	0.41–2.46	1.06	0.39–2.86
	Non-abdominal	4	0.51	0.18–1.46	0.36	0.12–1.08
PNET	Abdominal	16	1.71	0.93–3.17	1.88	0.92–3.83
	Non-abdominal	10	0.82	0.40–1.66	0.81	0.83–1.69
Ependymoma^c^	Abdominal	3	1.01	0.34–2.98	—	—
	Non-abdominal	6	1.16	0.47–2.87	—	—
Controls	Abdominal	48				
(*n*=512)	Non-abdominal	63				

CBT=childhood brain tumour; CI=confidence interval; OR=odds ratio; PNET=primitive neuroectodermal tumour.

a Adjusted for maternal age, parity, multiple birth, mother born in a Nordic country, gestational age at birth, mode of delivery, breech position, birth weight, birth head circumference, level of hospital and hypertension during pregnancy.

b Includes the subtypes in the table, germ cell tumours and other miscellaneous tumours. Only subjects with information on all variables adjusted for are included.

c Multivariate analyses could not be performed because of the low number of cases.
